# Energy minimization segmentation model based on MRI images

**DOI:** 10.3389/fnins.2023.1175451

**Published:** 2023-04-14

**Authors:** Xiuxin Wang, Yuling Yang, Ting Wu, Hao Zhu, Jisheng Yu, Jian Tian, Hongzhong Li

**Affiliations:** ^1^Chongqing University of Posts and Telecommunications, Chongqing, China; ^2^Institute for Advanced Sciences, Chongqing University of Posts and Telecommunications, Chongqing, China; ^3^School of Civil Engineering, Guangdong Communication Polytechnic, Guangzhou, China

**Keywords:** image segmentation, MRI, anatomical atlas, lesions filling, energy

## Abstract

**Introduction:**

Medical image segmentation is an important tool for doctors to accurately analyze the volume of brain tissue and lesions, which is important for the correct diagnosis of brain diseases. However, manual image segmentation methods are time-consuming, subjective and lack of repeatability, it needs to develop automatic and reliable methods for image segmentation.

**Methods:**

Magnetic Resonance Imaging (MRI), a non-invasive imaging technique, is commonly used to detect, characterize and quantify tissues and lesions in the brain. Partial volume effect, gray scale in homogeneity, and lesions presents a great challenge for automatic medical image segmentation methods. So, the paper is dedicated to address the impact of partial volume effect and multiple sclerosis lesions on the segmentation accuracy in MRI. The objective function of the improved model and the post-processing method of lesion filling are researched based on the fuzzy clustering space and energy model.

**Results:**

In particular, an energy-minimized segmentation algorithm is proposed. Through experimental verification, the AR-FCM algorithm can better overcome the problem of low segmentation accuracy of the RFCM algorithm for tissue boundary voxels and improve the segmentation accuracy of this algorithm. Meanwhile, a multi-channel input energy-minimization segmentation method with lesion filling and anatomical mapping is further proposed.

**Discussion:**

The feasibility of the lesion filling strategy using post-processing can be confirmed and the segmentation accuracy is increased by comparison experiments.

## 1. Introduction

In recent years, brain diseases are becoming more and more dangerous to human health, and their prevention and treatment are gradually becoming the most important concerns in the medical field. The number of patients with brain diseases such as cerebral thrombosis, cerebral infarction and multiple sclerosis accounts for 30% of the total number of human diseases (Feigin et al., 2021). Multiple sclerosis (MS) is one of the chronic autoimmune diseases of the central nervous system. It is characterized by the demyelination of axons in the cerebral cortex and other gray matter (GM) and white matter (WM) regions, forming focal inflammatory lesions accompanied by the production of symptoms such as pain, impaired mobility, and poor vision (Lassmann, 2018). According to research, the incidence of this disease has been increasing worldwide, and the number of patients with multiple sclerosis currently exceeds more than 2 million cases (Browne et al., 2014). In research and clinical practice, magnetic resonance imaging (MRI) is often used as the most important tool for the diagnosis of MS because of its high sensitivity, good imaging quality, and low radiation output, which can well detect MS plaques and quantify the number and volume of lesions (Filippi et al., 2016). In general, MRI sequences are divided into different categories, including T1-weighted, T2-weighted, proton density (PD), and fluid attenuation inversion recovery (FLAIR). MS lesions usually appear as areas of low signal intensity (low signal to normal white matter) on T1-weighted images and focal areas of high signal intensity (high signal) on T2-weighted images, reflecting tissue water content. Except for cerebrospinal fluid (CSF) suppression, FLAIR images have similar features to T2-w images (Poser et al., 1983). The identification of the number and volume of MS lesions is a critical process in diagnosis, and for the presence of white matter lesions, they are usually depicted manually by specialists in hospitals (Wang et al., 2019). However, manual segmentation of MS lesions is very time consuming and there is a large variation in the depiction of different experts (Gva et al., 2020), in contrast, automated segmentation of MS lesions can save time and reduce the dependence on the observer. However, the presence of grayscale unevenness and noise in MRI, among others (Bajracharya and Rawal, 2015), make accurate segmentation a challenge.

Image segmentation is a common method for extracting tissues such as white matter, gray matter, and CSF from MRI images for quantitative brain tissue analysis (Dora et al., 2017). Over that last decades, many researchers focus on medical image segmentation, which has led to the rapid development of medical image techniques. Brain tissue segmentation methods can be broadly classified into five categories: manual segmentation, region-based segmentation methods, threshold-based segmentation methods, clustering-based segmentation methods, and methods with feature extraction and classification (Jiang et al., 2022). In brain tissue segmentation, clustering methods are statistical techniques based on pixels or voxels and are usually processed for T1-weighted MR images. Among the clustering algorithms based on minimization objective functions, the most theoretically sound and most applied clustering method is the Fuzzy C-Means (FCM) algorithm. The FCM algorithm was proposed by Dunn et al. Although it has better segmentation performance than hard clustering methods, it has poor noise immunity and does not segment noisy MR images well (Tian et al., 2021). To reduce the sensitivity of the FCM algorithm to noise, Pham proposed a new objective function for adding spatial context to the fuzzy c-mean algorithm (Pham, 2001). Its objective function includes a penalty term, which is similar to the Markov random field prior, and is consistent with the desired behavior of the affiliation function determined by the values of the fuzzy factor parameters thus improving it compared to the FCM algorithm, but is more sensitive to the boundaries of the organization (Dobson and Giovannoni, 2019). However, these classical segmentation methods also face some challenges when dealing with images in the presence of lesions, as the intensity of the lesion portion is usually similar to that of normal tissue (Zhao et al., 2018).

In order to handle brain MRI that contain both grayscale unevenness, noise, and MS focal regions, this paper presents anatomical mapping based on the RFCM algorithm, as well as a focal filling strategy using post-processing, which is applied to segment normal brain tissue on brain MRI images suffering from MS. It is demonstrated that the improved RFCM algorithm strategy improves the accuracy of brain MRI image segmentation.

## 2. Methods

### 2.1. Atlas Robust Fuzzy C-mean algorithm

**Figure 1:**
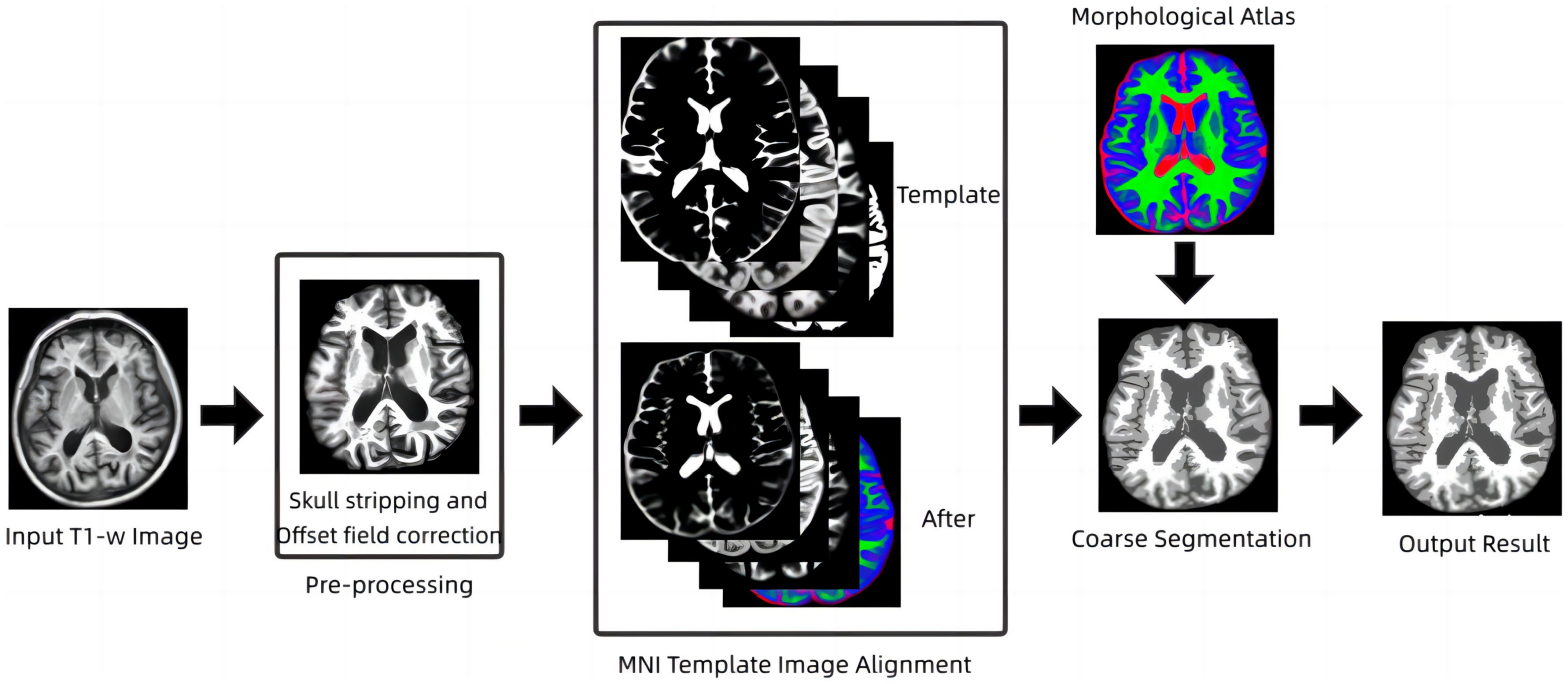
Flowchart of energy minimization algorithm based on anatomical mapping.

The fuzzy clustering space model is an earlier method that uses penalty terms to achieve smoothing of images without being too sensitive to noise, but the model is less effective for segmentation of tissue boundary parts, which is due to the volume effect that can exist in magnetic resonance images, and the volume effect causes the boundaries of brain structures in images to become discontinuous and unclear (Wang et al., 2018). To address this problem, an energy minimization algorithm based on anatomical mapping is proposed in this paper. The model links fuzzy clustering and statistical probability probability mapping by constructing a constraint term in the objective function of the fuzzy clustering space model, called the AR-FCM algorithm (Atlas Robust FCM, AR-FCM). This model inherits the advantages of the fuzzy clustering space model, uses statistical probability mapping to constrain the segmentation of brain tissue, and uses morphological mapping to redistribute voxels at tissue boundaries after segmentation to reduce the effect of partial volume effects. The flow of the method is shown in Figure 1 below.

### 2.2. S-Lesion Filling algorithm

From an image processing perspective, MS lesions can affect tissue segmentation, causing GM and WM to be classified in the wrong category. MS lesions may affect the estimation of segmentation parameters, leading to changes in tissue boundaries (Ma et al., 2010; Prados et al., 2016), which can affect subsequent morphological studies, including atrophy measurements, tissue volume measurements, etc. Therefore, lesion filling is needed to reduce the negative impact that MS lesions may have on image analysis in order to improve tissue segmentation accuracy (Tian et al., 2022). Briefly, the lesion filling process uses WM image intensities to synthetically estimate filled WM lesions.

**Figure 2:**
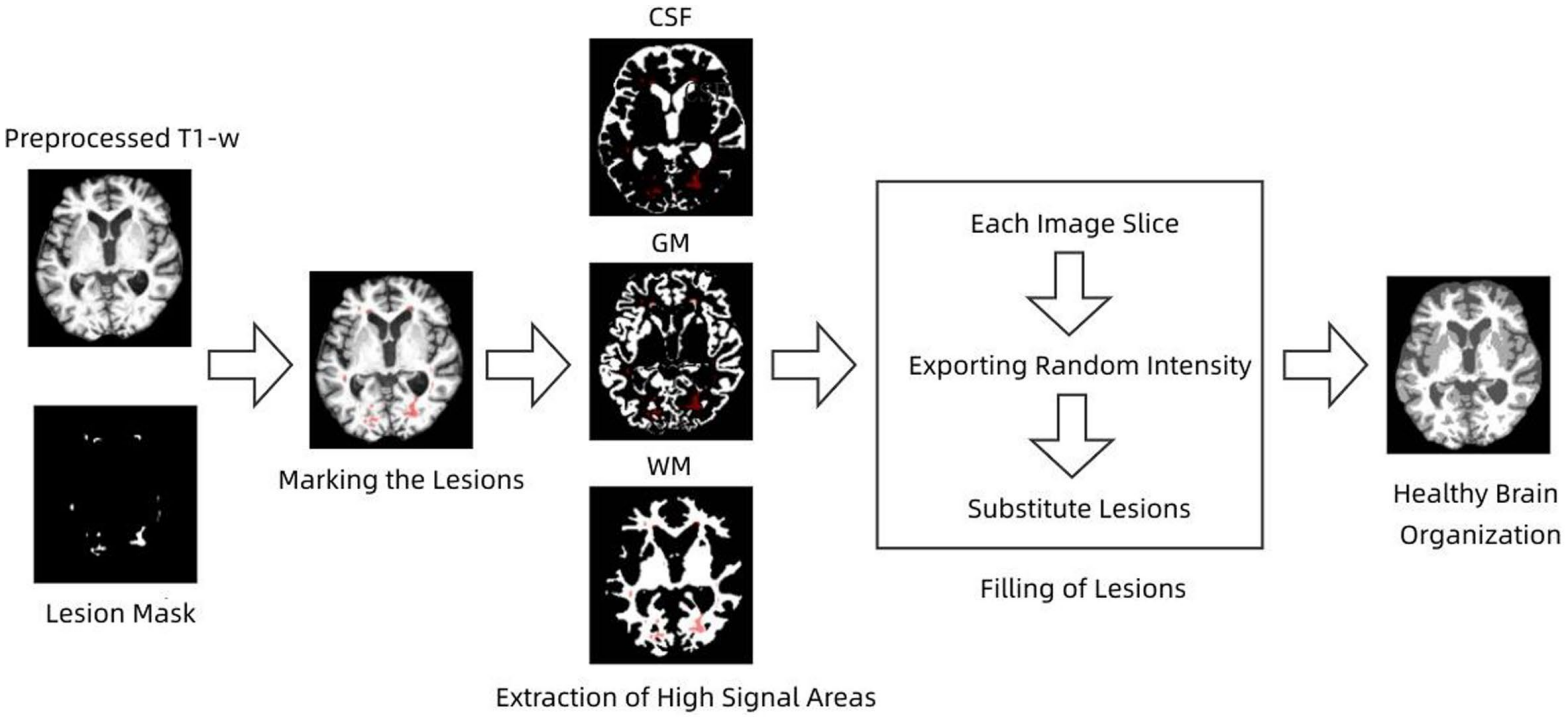
Flow chart of lesion filling.

S-Lesion Filling (SLF) algorithm is a combined global and local method for filling WM lesions (Valverde et al., 2014; Makropoulos et al., 2018). The filling process of the lesions was performed by taking each axial slice that constituted the 3D image and calculating the mean and standard deviation of the NAWM tissue signal intensity. The calculated mean and standard deviation values are used to generate a normal distribution with a mean value equal to the calculated NAWM mean intensity and a standard deviation equal to half of the calculated NAWM standard deviation. The standard deviation was always fixed to half of the WM mean, independent of the data set used, and this value was chosen empirically to balance the accuracy of the method for 1.5 and 3 T images. The lesion voxel intensities of the current image slice were then replaced by random values of the generated distribution. The process is repeated until all image slices are completed. The flow of the algorithm is shown in Figure 2.

### 2.3. Lesion Filling and Atlas RFCM algorithm

**Figure 3:**
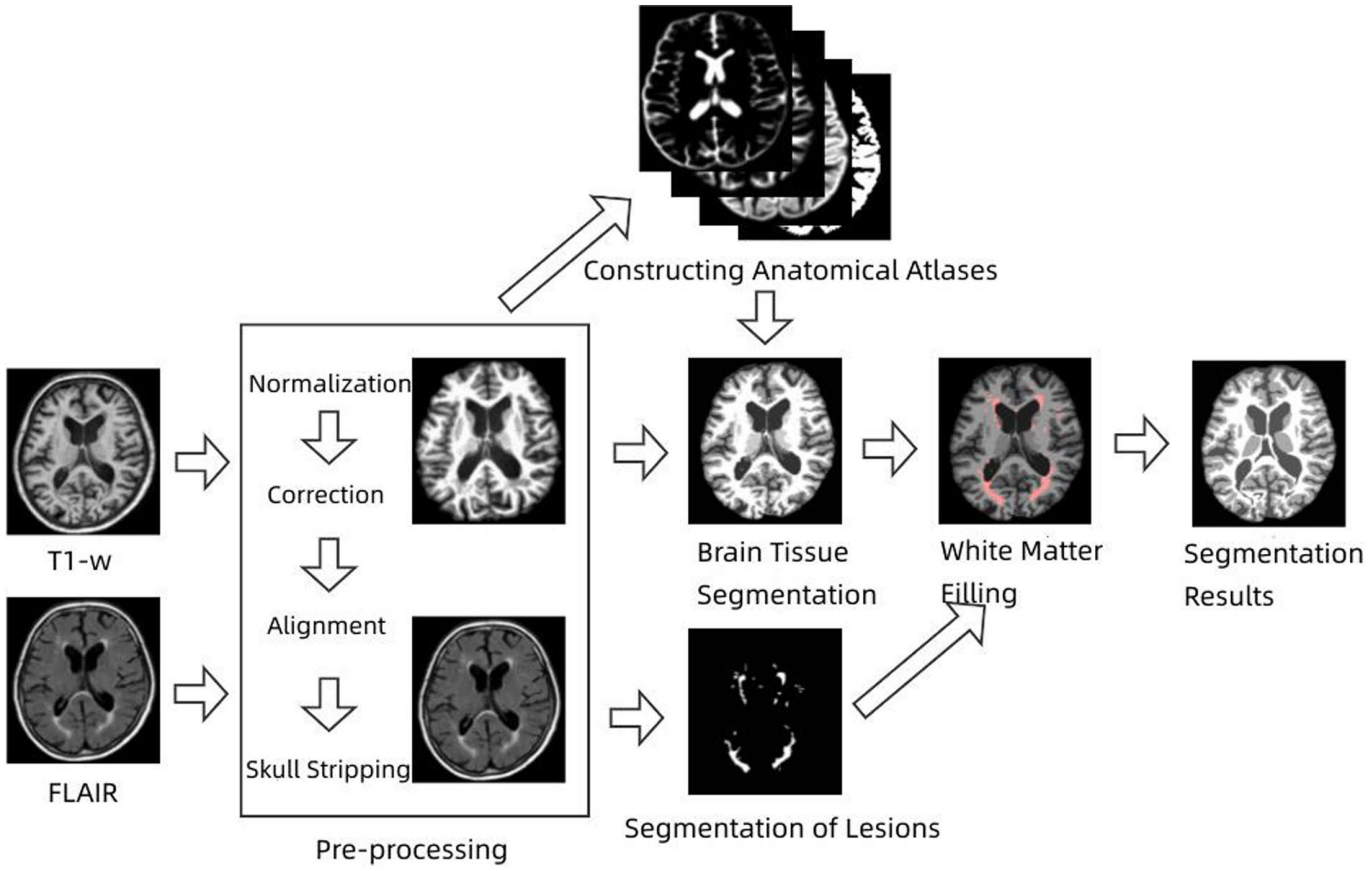
Flow chart of energy minimization algorithm based on lesion filling and anatomical mapping.

Pathophysiological studies have shown that conventional magnetic resonance imaging has limited sensitivity to small structural changes, both in lesions and normal gray and white matter (Fan et al., 2016; Weiskopf et al., 2021). This suggests that quantitative volumetric analysis of brain tissue and lesions directly from MRI images is not feasible and therefore requires extraction of brain structures such as cerebrospinal fluid, white matter, gray matter, and lesions using image segmentation techniques prior to quantitative analysis (Wang et al., 2014). Multiple sclerosis lesions are an autoimmune neurodegenerative disease whose main feature is the presence of white matter lesions (WM Lesion, WML), which are damaged white matter tissues associated with increased CSF levels. Some classical segmentation methods also face some challenges when dealing with images with lesions, because the intensity of the lesion part is often similar to that of normal tissue and the AR-FCM algorithm proposed in this paper does not segment the brain tissue with lesions well. In general, MS lesions in FLAIR sequences are less severe than CSF, exhibit high signal abnormalities in GM, and can be identified based on contrast (Pohl et al., 2007; Fransen et al., 2020). Based on this feature, lesion areas can be processed using focal filling prior to segmentation, effectively reducing misclassification of CSF and white matter tissue. According to this strategy, an energy minimization algorithm based on lesion filling and anatomical mapping, namely the LFA-FCM algorithm (Lesion Filling and Atlas RFCM), is proposed in this paper. The method requires input T1-w images and FLAIR images, screening out focal regions on FLAIR images using the segmentation lesion method, and then using lesion filling to fill in and replace abnormal values in T1-w images to construct healthy brain MRI images, and finally completing the segmentation using the AR-FCM method. The flow of the method is shown in Figure 3.

### 2.4. Evaluation indicators

Three commonly used evaluation metrics are selected to measure the segmentation results of this method and other methods, namely Dice similarity coefficient (DSC), Volumetric similarity (*VS*), and Hausdorff distance (HD). These three metrics are chosen because the Dice coefficient is sensitive to the internal organization of the segmentation, while the Hausdorff distance is sensitive to the boundaries of the segmentation, and the volumetric similarity shows the overall segmentation effect.

The Dice similarity coefficient is an ensemble similarity measure function that is widely used to calculate the similarity of two samples and takes values in the range of [0,1]. In image processing, the Dice coefficient is mainly used to measure the accuracy of segmentation within a tissue. The Dice similarity coefficient is calculated by both the gold standard image (GT) and the computational segmentation mask (SEG) as follows:


(1) $$DSC=\frac{2\left|SEG\cap GT\right|}{\left|SEG\right|\cup \left|GT\right|}$$

The closer the Dice similarity coefficient is to 100 indicates that the segmentation results are closer to those of the expert manual segmentation. To make the results more accurate, the DSC value is multiplied by 100 in this paper.

Hausdorff distance is a measure describing the degree of similarity between two sets of points, and it is a defined form of distance between two sets of points. It is mainly used in image segmentation to measure the segmentation accuracy of the boundary. It is calculated by the distance (95th percentile) between the segmentation points in the gold standard image and the segmentation points in the segmentation mask as follows:


(2) $$HD=\underset{p\;\;\in \;\;SEG_{c}}{\max}\underset{p^{\prime }\;\;\in \;\;GT_{c}}{\min}\left|p-p^{\prime }\right|$$

The closer the Hausdorff distance is, the better the segmentation is indicated.

The volume similarity is also calculated by both the gold standard image and the computed segmentation mask, and the more the value converges to 100, the better the segmentation effect:


(3) $$VS=1-\left|\frac{GT-SEG}{GT+SEG}\right|$$

## 3. Results

In this paper, two sets of experiments were conducted, and the first set of experiments was selected to compare the AR-FCM algorithm with the RFCM algorithm, and the segmentation categories were set to three categories: CSF, gray matter and white matter, and the parameter settings of the two methods are shown in Table 1 below. The second group of experiments is to use RFCM algorithm, AR-FCM algorithm and LFA-FCM algorithm for brain tissue segmentation respectively, and the parameter settings of the three methods are shown in Table 2 below. The parameters of the AR-FCM algorithm as well as the LFA-FCM algorithm were required to be consistent in the experiment, which was to verify whether the strategy of increasing lesion filling could improve the accuracy of tissue segmentation with the same parameters. Two experiments were done on the MICCAI 2018 MRI brain segmentation challenge data set, and the hardware platform and software used for the experiments are shown in Table 3.

**Table 1:** RFCM and AR-FCM parameter setting table.

Methods	*q*	*γ*	*w*	*β*	*n*	*thr*
RFCM	2	-	-	1	500	0.001
AR-FCM	2	0.025	1	-	200	0.001

**Table 2:** Parameter settings for RFCM, AR-FCM and LFA-FCM methods.

Methods	*q*	*γ*	*w*	*β*	*n*	*thr*
RFCM	2	–	–	1	500	0.001
AR-FCM	2	0.025	1	–	200	0.001
LFAR-FCM	2	0.025	1	–	200	0.001

**Table 3:** Table of experimental software and hardware parameters.

Category	Parameters
Operating system	Windows 10
CPU	Intel(R) Core(TM) i5-9400F CPU 2.90GHz
RAM	16GB
Simulation software	Matlab 2019b

**Figure 4:**
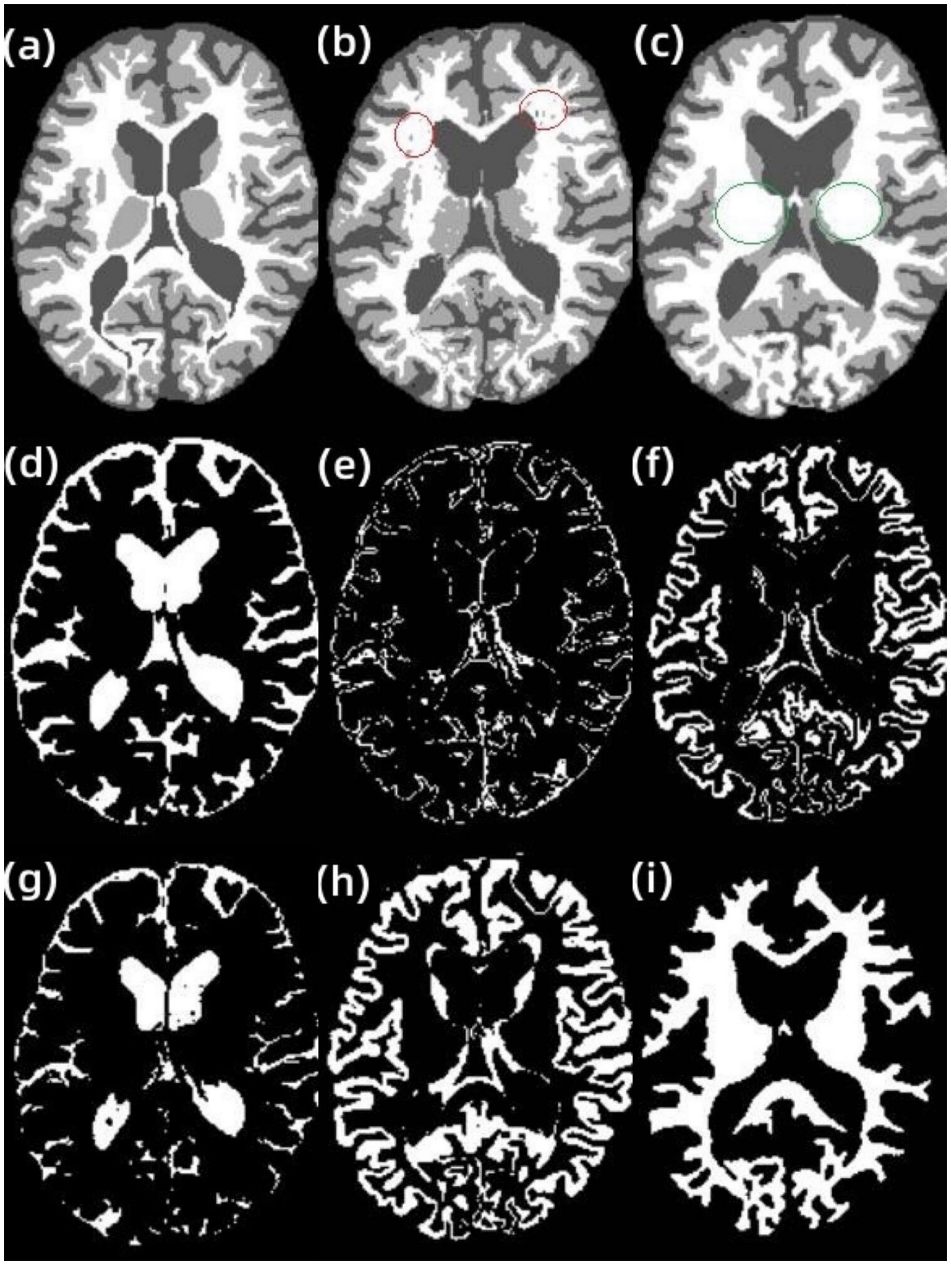
Segmentation results of AR-FCM and RFCM algorithms. **(A)** gt image, **(B)** AR-FCM, **(C)** RFCM, **(D)** cerebrospinal fluid, **(E)** gray matter, **(F)** white matter, **(G)** cerebrospinal fluid, **(H)** gray matter, and **(I)** white matter.

In Figure 4A, shows the original image, and Figure 4B,C show the results of AR-FCM and RFCM algorithm segmentation. Comparing the gold standard image and the result image obtained by the two algorithms, observing the part of the image (c) circled by green circles, we can see that many gray matter parts of the RFCM algorithm are divided into white matter, which leads to too low accuracy of gray matter segmentation and too high accuracy of white matter segmentation. On the contrary, the gray matter around CSF was preserved because AR-FCM used morphological maps to re-divide part of the volume of tissue after tissue segmentation. The second column from left to right is the result of segmentation of cerebrospinal fluid, gray matter and white matter for RFCM, and the third column from left to right is the result of segmentation of cerebrospinal fluid, gray matter and white matter for AR-FCM. The comparison between the second and third columns above shows that RFCM is less effective than AR-FCM in segmenting gray and white matter. Moreover, AR-FCM is better for the tissue segmentation between the boundaries and retains more details, which is achieved by using morphological mapping to re-divide some of the volume regions. The method in this paper improves the segmentation accuracy of the RFCM algorithm and requires only fewer iterations than the RFCM algorithm. However, the part circled in red in image (b) is incorrectly segmented as gray matter tissue, which is the presence of focal tissue in the data set used. It can be seen that the AR-FCM algorithm also does not segment the brain tissue with the presence of lesions better.

**Figure 5:**
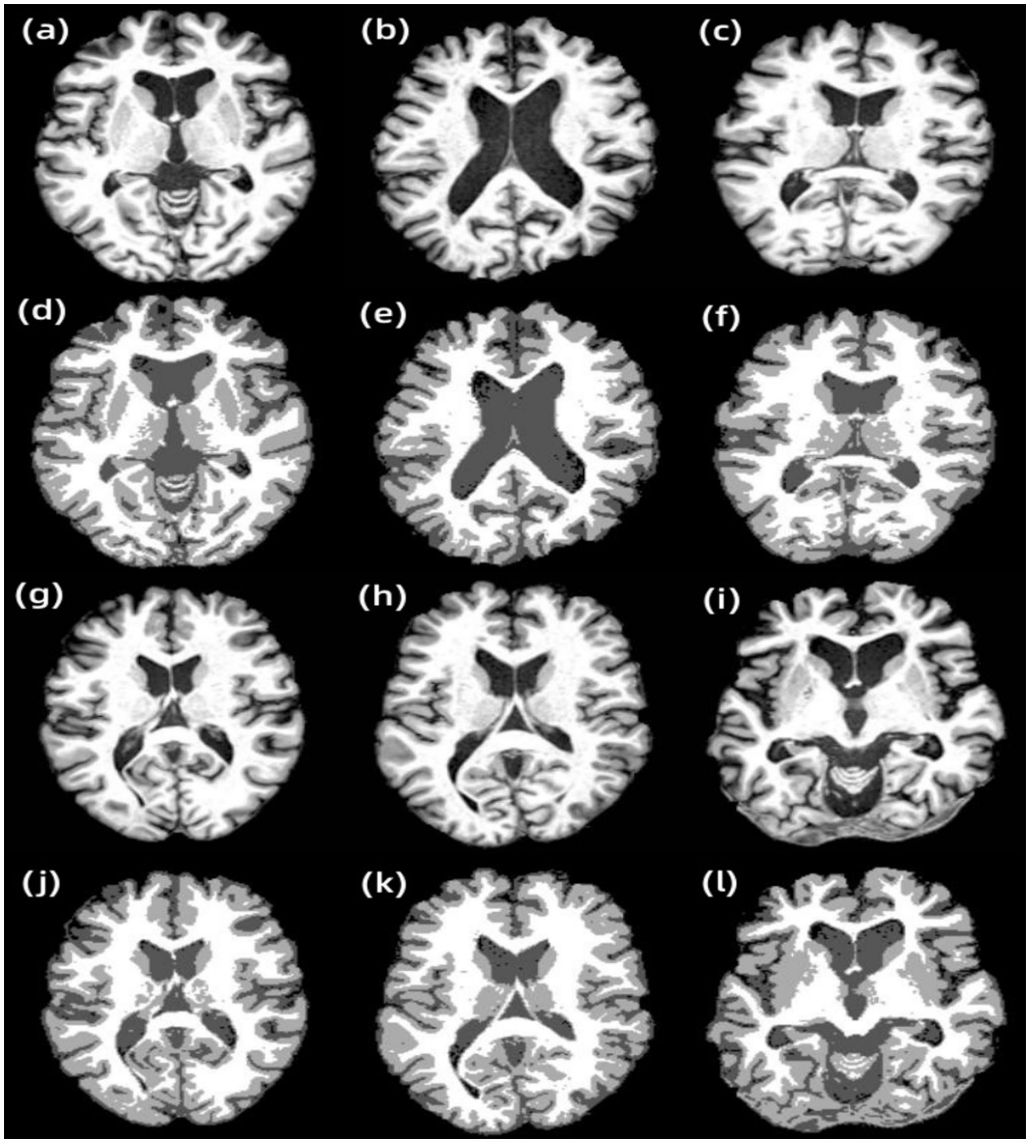
Results of other examples of AR-FCM algorithm segmentation. **(A)** Subject 1, **(B)** subject 2, **(C)** subject 3, **(D)** subject 1 segmentation result, **(E)** subject 2 segmentation result, **(F)** subject 3 segmentation result, **(G)** subject 4, **(H)** subject 5, **(I)** subject 6, **(J)** subject 4 segmentation result, **(K)** subject 5 segmentation result, and **(L)** subject 6 segmentation result.

In addition, other examples in the data set are processed in this paper using the AR-FCM algorithm. Figure 5 shows the results of applying the energy-minimization segmentation algorithm based on anatomical mapping to other examples in the data set. The first and third rows are the T1-w images of the subject, and the second and fourth rows are the results of segmentation using the AR-FCM algorithm. The black part is CSF, the light gray part is gray matter, and the white part is white matter. Comparing the T1-w images with the segmented result images, it can be seen that the segmented parts of the brain tissue are more consistent with the structures shown in the T1 images. In addition, this method is stable and fully automated, which can yield satisfactory results in practical applications.

**Figure 6:**
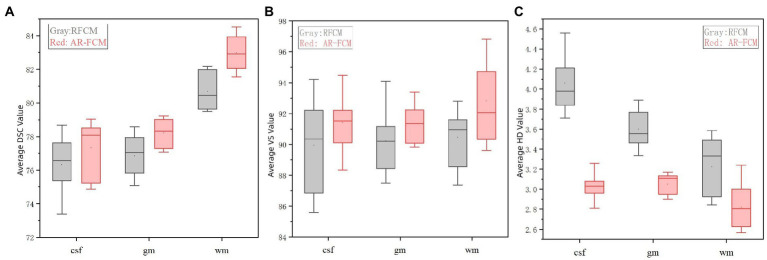
Indicator data visualization results. **(A)** Average DSC values of AR-FCM and RFCM, **(B)** average *VS* values of AR-FCM and RFCM, and **(C)** average HD values of AR-FCM and RFCM.

**Figure 7:**
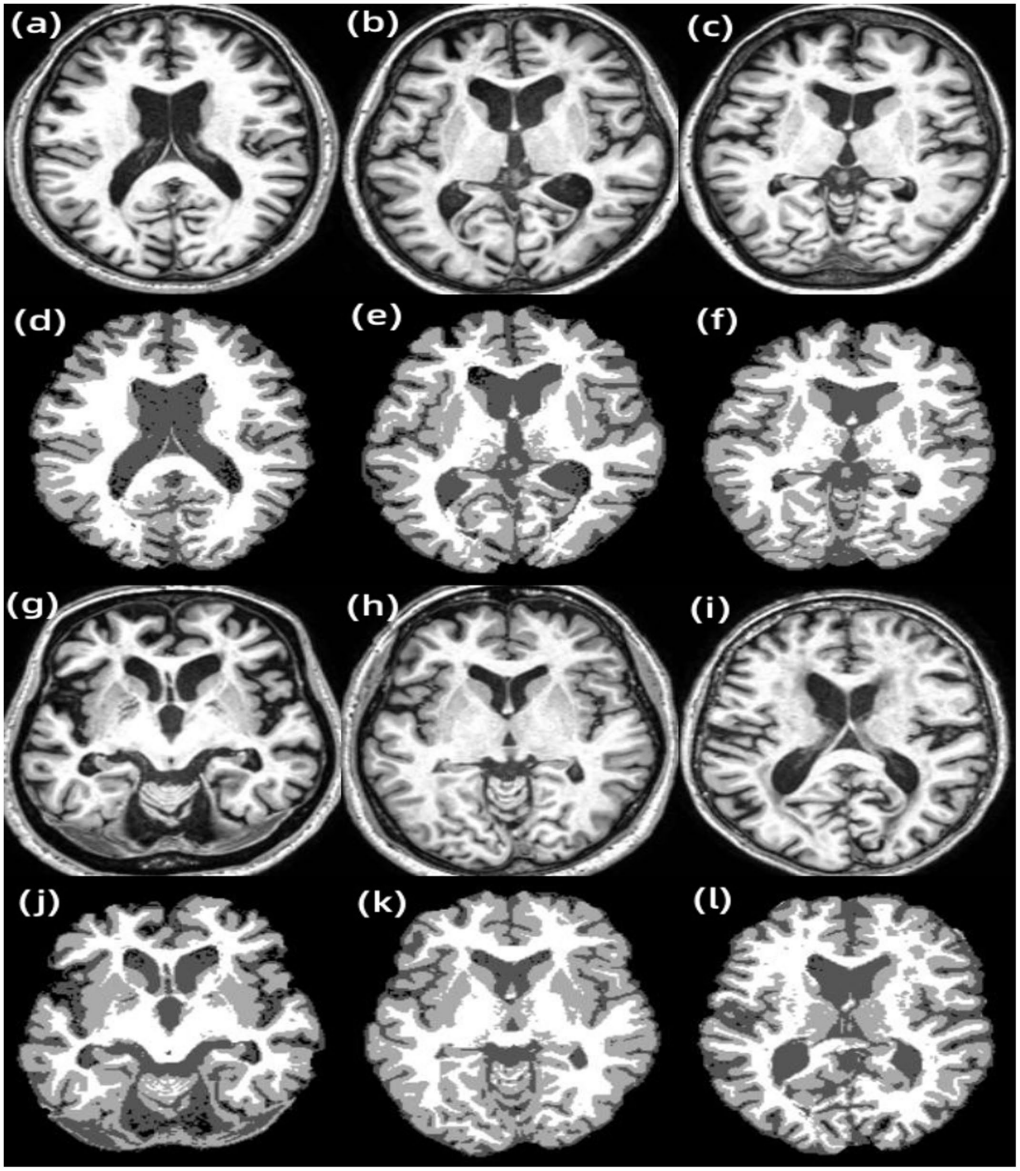
Results of other examples of LF-ARFCM algorithm segmentation. **(A)** Subject 1, **(B)** subject 2, **(C)** subject 3, **(D)** subject 1 segmentation result, **(E)** subject 2 segmentation result, **(F)** subject 3 segmentation result, **(G)** subject 4, **(H)** subject 5, **(I)** subject 6, **(J)** subject 4 segmentation result, **(K)** subject 5 segmentation result, and **(L)** subject 6 segmentation result.

Table 4 shows the mean DSC, *VS* and HD values obtained by the RFCM algorithm for each subject, and Figure 6 show the results of the data visualization in the table, respectively. By comparing the average DSC, *VS*, and HD values obtained by the two clustering algorithms, AR-FCM and RFCM, the overall data shows that the average, as well as the values obtained by the AR-FCM algorithm are higher than those of the RFCM algorithm, which indicates that the overall performance of the AR-FCM algorithm is better than that of the RFCM algorithm. The red box plots in Figure 6A, through image (c), represent the AR-FCM segmentation results, and the gray box plots represent the RFCM segmentation results. It is found that both algorithms have higher segmentation accuracy for white matter compared to other tissues, while the AR-FCM algorithm has higher average DSC values for CSF, gray matter, and white matter, which indicates that the AR-FCM algorithm is more accurate for segmenting voxels within brain tissue, which is related to the statistical probability mapping as a constraint. The results in Figure 7 show that the AR-FCM algorithm segmented all three tissues to obtain higher Hausdorff distance values than the RFCM algorithm, and the AR-FCM algorithm segmented each tissue to obtain a minimum HD value greater than the RFCM algorithm obtained a maximum HD value. It indicates that the AR-FCM algorithm is more accurate for segmentation of boundaries, and verifies the feasibility of the strategy of post-processing and re-dividing some volume regions using morphological spectrograms in this paper. In terms of *VS* scores, the difference between the AR-FCM algorithm and the RFCM algorithm for CSF and GM tissue segmentation is not significant, but the AR-FCM algorithm has improved the average *VS* score for WM. In conclusion, AR-FCM is better than RFCM for segmentation of brain tissues in MRI images.

**Table 4:** Average DSC, *VS* and HD values for AR-FCM and RFCM.

Models	Indicators	CSF	GM	WM
RFCM	DSC	76.33 ± 2.94	76.86 ± 1.78	80.67 ± 1.51
	*VS*	89.95 ± 4.36	90.22 ± 3.87	90.46 ± 3.23
	HD	4.06 ± 0.53	3.59 ± 0.27	3.23 ± 0.36
AR-FCM	DSC	77.32 ± 2.09	78.21 ± 1.01	82.98 ± 1.56
	*VS*	91.41 ± 3.07	91.34 ± 2.06	92.83 ± 4.11
	HD	3.03 ± 0.23	3.05 ± 0.12	2.81 ± 0.42

**Figure 8:**
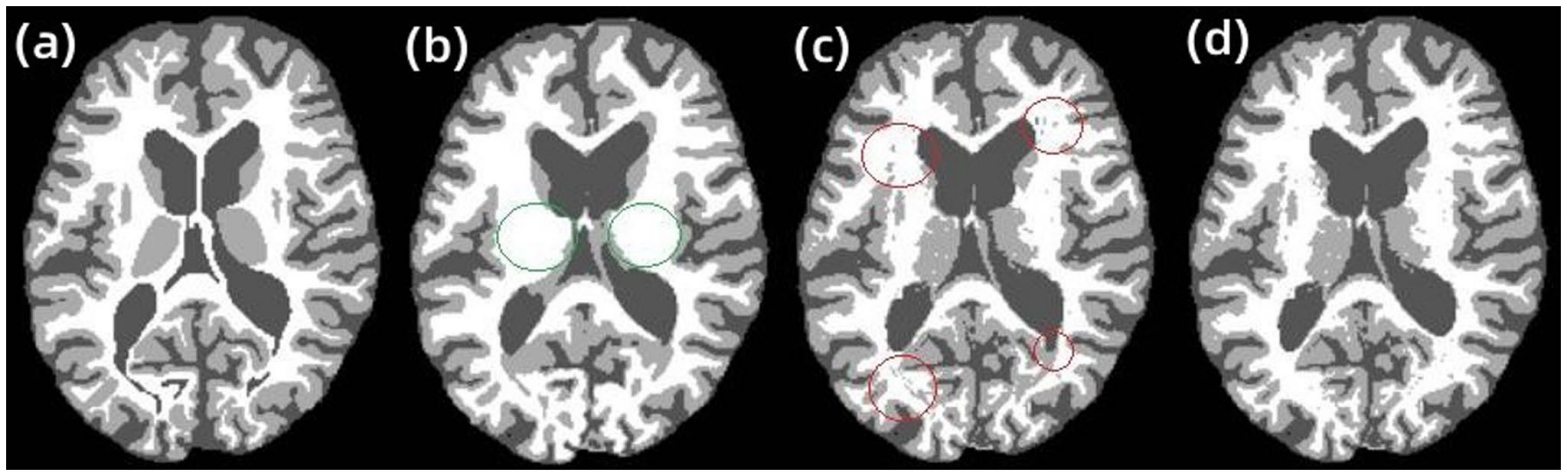
RFCM, AR-FCM, and LFA-FCM segmentation results. **(A)** gt, **(B)** RFCM, **(C)** AR-FCM, and **(D)** LFA-FCM.

The results of the second group experiments with RFCM, AR-FCM and LFA-FCM are shown in Figure 8A. shows the gold standard image provided in the dataset, Figure 8B–D show the brain tissue segmented using RFCM, AR-FCM, and LFA-FCM, respectively. Comparing image (a) with image (b), the regions circled in green belong to gray matter tissue in the gt image, while the RFCM algorithm classifies all these regions as white matter, which will result in large volume measurements of white matter tissue and small volume measurements of gray matter tissue. Observe image (c) and image (d), the regions circled in red in (c), which belong to white matter tissue in the gt image, and the AR-FCM algorithm incorrectly divides these regions into gray matter tissue. Image (d) shows the result of improved segmentation by the AR-FCM algorithm using lesion filling. It can clearly be seen that the incorrectly segmented gray matter tissue in image (c) is correctly segmented into normal white matter tissue after processing using focal filling, which indicates that the segmentation accuracy of white matter tissue with gray matter tissue can be improved using the post-processing focal filling strategy.

Figure 7 shows the segmentation results of other examples in the LFA-FCM algorithm segmentation dataset. The first and third rows are the T1-w images of the subjects, and the second and fourth rows are the results of segmentation using the LFA-FCM algorithm. The black part is the CSF, the light gray part is the gray matter, and the white part is the white matter. From the above figure, it can be seen that the method segmented the brain tissue better, and it is almost consistent with the brain tissue structure demonstrated by the T1-w images.

**Figure 9:**
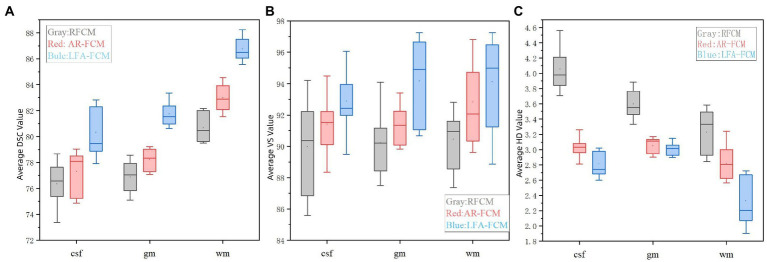
Indicator data visualization results. **(A)** Average DSC values of RFCM, AR-FCM and LFA-FCM, **(B)** average *VS* values of RFCM, AR-FCM and LFA-FCM, and **(C)** average HD values of RFCM, AR-FCM and LFA-FCM.

The results of segmentation by the three methods were quantitatively analyzed. The results of the three methods to obtain the three index scores are shown in Table 5, and Figure 9 show the results of data visualization in the table, respectively. By the results shown, Figure 9A, LFA-FCM obtained the highest DSC scores, especially the DSC values of segmented white matter tissue and gray matter tissue, and the worst results obtained in all seven sets of images processed were better than the best performance obtained by the other two methods. This is directly related to the operation of adding white matter filling before segmentation, indicating that lesion filling can effectively reduce the effect of T1-w multiple sclerosis lesions with low signal intensity on automatic brain tissue segmentation, thus improving the segmentation accuracy of the segmentation algorithm for white and gray matter tissues. As seen in Figure 9B, the LFA-FCM algorithm is an overall improvement in the accurate measurement of the volume of each part of the brain tissue. Figure (c), shows the average HD values obtained by the three algorithms, and although the average Hausdorff distance calculated by RFCM is closer, the overall result is still worse than the performance of the other two methods. In particular, the CSF tissue is far better than on the other two methods. In Figure 9C, the difference between the average Hausdorff distance calculated by the two methods, AR-FCM and LFA-FCM, is not significant, indicating that the white matter filling algorithm is not effective in improving the border tissue segmentation.

**Table 5:** Average DSC, *VS* and HD values for RFCM, AR-FCM and LFA-FCM.

Models	Indicators	CSF	GM	WM
RFCM	DSC	76.33 ± 2.94	76.86 ± 1.78	80.67 ± 1.51
	*VS*	89.95 ± 4.36	90.22 ± 3.87	90.46 ± 3.23
	HD	4.06 ± 0.53	3.59 ± 0.27	3.23 ± 0.36
AR-FCM	DSC	77.32 ± 2.09	78.21 ± 1.01	82.98 ± 1.56
	*VS*	91.41 ± 3.07	91.34 ± 2.06	92.83 ± 4.11
	HD	3.03 ± 0.23	3.05 ± 0.12	2.81 ± 0.42
LFA-FCM	DSC	80.32 ± 2.53	81.73 ± 1.72	86.77 ± 1.46
	*VS*	92.88 ± 2.36	94.17 ± 3.08	94.11 ± 3.13
	HD	2.81 ± 0.21	3.01 ± 0.12	2.33 ± 0.43

## 4. Discussions

An anatomical atlas-based energy minimization algorithm (Atlas Robust FCM) is firstly proposed for problems such as offset fields and lesions in brain image segmentation. In the objective function of a fuzzy clustering space model, the constraints are constructed to link the fuzzy clustering and the statistical probability graph. Statistical probability mapping is used as a constraint to limit the over-segmentation of brain tissue. After the brain tissue is segmented, the voxels of the volume part are redistributed using morphological mapping, which resolves the unclear and discontinuous boundary of the target structure. It will lead to the problem that the model of fuzzy clustering space is not accurate when organize the boundary region of classification. The AR-FCM clustering algorithm is verified by comparison experiments to overcome the problem of low accuracy of RFCM clustering algorithm for boundary tissue segmentation and improve the segmentation accuracy of RFCM algorithm.

An energy minimization algorithm (Lesion Filling and Atlas FCM) based on lesion filling and anatomical mapping was proposed for brain tissues with lesions that were not well segmented by the AR-FCM algorithm. Based on the feature that MS lesions in FLAIR sequences are lighter than CSF and exhibit high signal abnormalities in GM, lesion regions are filtered out on FLAIR images using the lesion segmentation method (SLF). The T1-w images were coarsely segmented using a clustered segmentation algorithm, and only the tissue with possible lesions was processed. Among the structures coarsely segmented out of T1-w, the part related to GM was selected and filtering conditions were set to filter out the overlapping region that met the conditions in the coarsely segmented brain tissue and the focal region. For this region, the abnormal values in the T1-w image are filled and replaced by using the lesion filling, and a healthy T1-w image is constructed, and finally the AR-FCM algorithm is used again to complete the segmentation. The average DSC, HD and *VS* scores of LFA-FCM are found to be higher than those of AR-FCM through comparison experiments, which indicates that the strategy of using post-processing lesion filling is indeed feasible and the segmentation accuracy is indeed improved.

Innovative improvements are made to solve the problems of partial volume effect, gray scale inhomogeneity, and sensitivity of the fuzzy clustering space model to tissue boundaries in magnetic resonance images. The specific improvements are based on the fuzzy clustering space model, using statistical probability mapping as a constraint term in the energy function to limit the over-segmentation of brain tissue, and after segmentation, using morphological mapping to reassign voxels between tissue boundaries; the energy minimum segmentation algorithm segments MRI brain images in the presence of white matter lesions, which may misjudge the focal regions and lead to the assessment of brain white matter volume inadequate. The specific improvement method uses the focal segmentation method to estimate the focal region on FLAIR images, screens out the lesioned tissue, and replaces the abnormal values by filling them using the focal filling method.

## 5. Conclusion

In this paper, the MRI brain image segmentation algorithm makes an intensive study, mainly considering the effects of offset field, cranial bone, volume effect and lesion on the segmentation results. A large number of MRI images are segmented and compared with existing related algorithms in terms of the effectiveness and accuracy of segmentation results. Experiments have verified that the algorithm proposed reduces the effects of partial volume effects and lesions; achieves accurate and efficient brain image segmentation by MRI. Therefore, it can better diagnose the brain disease, manage the patients effectively in the early stage and reduce the possibility of the brain disease worsening.

## Data availability statement

The original contributions presented in the study are included in the article/supplementary material, further inquiries can be directed to the corresponding authors.

## Author contributions

XW contributed to the experiments design and conception. TW, HZ, and JY performed experiments and data analysis. YY, JT, and HL contributed to data analysis. All authors edited the manuscript.

## Funding

This work was sponsored in part by the National Natural Science Foundation of China (Grant No. 61605021); in part by Science and Technology Research Program of Chongqing Municipal Education Commission (Grant No. KJQN202000624); in part by Opening Foundation of Key Laboratory of Opto-technology and Intelligent Control (Lanzhou Jiaotong University), The Ministry of Education (KFKT 2020–08); and in part by the eleventh Chongqing fledgling Eagle Project (No. CY220617).

## Conflict of interest

The authors declare that the research was conducted in the absence of any commercial or financial relationships that could be construed as a potential conflict of interest.

## Publisher’s note

All claims expressed in this article are solely those of the authors and do not necessarily represent those of their affiliated organizations, or those of the publisher, the editors and the reviewers. Any product that may be evaluated in this article, or claim that may be made by its manufacturer, is not guaranteed or endorsed by the publisher.
